# Monthly Periscope

**Published:** 1853-08

**Authors:** 


*Monthly Periscope.*—Dr. P. H. Strong, at a recent meeting of the Buff. City Med. Association, detailed a case of cholera infantum, in which the usual treatment being of no avail, and death being imminent, he resorted to the subnitrate of bismuth, in doses unusually large. The patient was an infant of one or two years of age. The quantity given was so large that an ounce lasted but two or three days, one-third of a tea-spoonful being given several times daily. Under these enormous doses, the child gained rapidly, and finally recovered. We hope for a full report from Dr. Strong on this subject. It is well known that bismuth is ranked by Orfila among the poisons, and that a leaden aspect around the eyes, sunken eyes, swollen and livid gums, and a tendency to passive hæmorrhage, are named among its poisonous effects. It seems from the researches of M. Lusanna, a Tuscan physician, that bismuth is soluble in the gastric juices, but is precipitated by the alkaline secretions of the duodenum. Its poisonous effects are attributable to its absorption into the circulation from the stomach. If, then, we precede its administration by magnesia, or other alkalies, we shall prevent its circulatory absorption, and send it down unchanged to the bowel to exercise its absorbent function. It is stated that it does *not* act as an irritant on the alimentary canal. The remedy is certainly worthy of a trial, and if we look upon it as an absorbent merely, it should be given in much larger doses than those usually prescribed.

There is a pleasant and a sensible article in the July number of Putnam's Monthly, entitled "Doctors." In a quiet and chatty style, the writer hits off some of the salient points in our craft very happily. We should like to give it entire, but can only segregate a few ideas which strike our fancy.

"There is no profession which depends so much for its efficiency on personal traits as that of medicine; for the utility of technical knowledge here is derived from individual judgment, tact, and sympathy. In other words, the physician has to deal with an unknown element. * * * *"

"Hence we associate a certain originality with the idea of a doctor; are apt to regard the vocation at the two extremes of superiority and pretension, and justly estimate the individuals of the class according to their capacity of insight and their principles of action, rather than by their mere acquisitions or rank as teachers. The uncertainty of medicine, as a practical art, thus induces a stronger reliance on individual endowments than is the case in any other liberal pursuit."

In another connection he says: "Accordingly there has sprung into existence, in our day, a personage best designated as the medical Jesuit; whose real vocation, as well as the process by which he acquires supremacy, fully justifies the appellation. Like his religious prototype, he operates through the female branches, who in their turn, control the heads of families; and the extent to which the domestic arrangements, the social relations, and even the opinions of individuals are thus regulated, is truly surprising." Many of our readers have felt this Jesuitism, and can appreciate the truthfulness of the description. After a handsome tribute to the philosophical and literary merit to be found in the profession, he describes Parisian medicine as the height of skill in diagnosis, with a great disregard to the knowledge and application of remedies.

"Accordingly the perfection of modern skill in the art, seems to result from an education in the French schools, combined with experience in English practice; thorough acquaintance with physiology and habits of acute observation, and accurate deduction are thus united to executive tact and ability."

An eminently sensible remark, it seems to us, and we find too, a capital hit, at a very common fault, in the following:

"There is a certain analogy between naval and medical men. Neither like to acknowledge the presence of danger."

Finally, illegitimate medicine is described as an offshoot of the transcendental spirit, which is invading the domains of science and art; "a return-to-nature-school, of which Wordsworth is the expositor in poetry, Fourier in social life, and Turner in painting." * * * "Somewhat of truth there is in this spiritualizing tendency of science, but Fact is the basis of all positive knowledge, and the most unwarrantable of all experiments, with only imagination for a guide, are those involving human life."

When a magazine like Putnam's (in our opinion best of all the monthlies) holds forth such sentiments to the secular world, we may hope for a resurrection of the lost dignity of medicine. And the secular press is the channel for such ideas. There they can reach the people; and so we say success to this writer in Putnam, and will include in our benediction good "Glauber Saultz, M. D.," whose autobiography is shaking the sides, and touching the hearts of the readers of the kindly "Knickerbocker."

*Asphyxia from Chloroform—Escalier's method of resuscitation.*—Prof. Rigaud details a case, in which a patient under chloroform suddenly stopped breathing; and the pulsation ceased. After two minutes spent in dashing cold water, and in using friction without avail, he resorted to the treatment of M. Escalier. "I introduced my right index finger into the mouth of the asphyxiated patient, made it pass along the base of the tongue, and lift the epiglottis. Then I pulled the tongue out of her mouth. This rapid motion caused an inspiration, which I made use of, to let her inhale ammonia. But as soon as I let go the tongue it slipped back, and the respiration ceased again. I repeated the same manœuvre, and produced again respiration. But this time I retained the tongue outside the mouth, and respiration continued to go on, after which, the woman recovered very soon, and all functions resumed their normal actions."

This simple account of a method which seems to us not unscientific, (inasmuch as it acts upon nerves sympathetically connected with the respiratory tract) may be of service. M. Nelaton attributes death from chloroform to syncope, and advises holding the patient up by the heels; urging that titillation of the nostrils, and friction to the extremities, must be absurd when the patient would not feel the pain of an amputation. M. Ricord looks upon it as asphyxia, and advises artificial respiration. He has thus rescued four persons from impending death. Escalier holds the same pathological views, and his method should be put in force, while making the preparations for artificial respiration.

*A substitute for Ergot.*—The N. O. Monthly Med. Register mentions an article from the pen of Dr. Washington, of Woodburn, Ky., in which he asserts the efficacy of dry cupping over different parts of the spinal cord, as a substitute for ergot in influencing uterine contraction. A cup applied over the origin of the sacral nerves for instance, is followed in a very short period by relaxation of the os uteri, and sphincter vaginæ. This accomplished, we may then transfer our cups to the lumbar, or lower dorsal region, and bring into action the expulsive forces. This is very convenient. By this simple means we have a perfect control of all the puckering strings, and can play the "open and shut game," to our heart's content. There is, perhaps, a little room for uncertainty in reasoning from cause to effect in this case. Very frequently we find the os uteri dilating just after some previous occurrence; and just so often, we find expulsive movement following the dilatation. It would be an easy matter to test this question. Find some patriotic female with a quiescent uterus, and appeal to her love of her species to submit to the necessary experimentation. There is no calculating on what a uterus at the ninth month of pregnancy will do; and the experiment should be conducted at some earlier period, when all is quiet.

But having no conveniences for experiment at hand, we propose to follow the argument of the Register on Dr. Washington's article. First, he says, if these views can be sustained by repetition of the experiment, it will go far to settle some disputed physiological questions. If cupping over the spinal axis causes uterine contraction, then the uterus is in part supplied with spinal nerves. He doubts the independence of the uterus over spinal control, so long conceded, admits that no spinal nerve has been directly traced to the uterus, but supposes that Dr. Robert Lee's discovery of a net-work of nerves, involving the uterus like the cordage of a balloon, must lead to some modifications of our previous views. This is a somewhat complex subject, and it is difficult to know what to believe. We think, however, that it *has* been anatomically demonstrated that the third and fourth sacral nerves, after entering the inferior hypogastric plexus, are distributed to the uterus. This would help Dr. Washington's theory, by establishing a connection with the spinal system. On the other hand, we do not see wherein Dr. Lee's discovery has any bearing. Dr. Lee does not demonstrate any spinal connection with his nervous net-work. Again, Mr. Beck demonstrates a precisely opposite fact; and distinguished anatomists have come to the conclusion, that the "great web" seen by Dr. Lee, is not nerve matter; and that the opinion that the nerves of the gravid uterus were enlarged proportionally with the organ itself, is untenable.

The present state of anatomical knowledge on this point, seems to be this: The uterus receives its nerves from the inferior hypogastric plexus of the ganglionic system — that, however, two spinal nerves at least, (the third and fourth sacral,) are also distributed to the uterus; and that finally the spinal axis so interweaves itself with the ganglionic nerves, as to render it difficult, and perhaps finally impossible, to estimate the amount of nerve force, sent from the spinal cord to the uterus.

It will be seen that this distribution does not present any insuperable anatomical objections to Dr. Washington's theory. It does, however, render it improbable, that so marked an effect can be produced on the uterus, by an impression so trivial on the spinal cord. Analogy increases this improbability. For all the abdominal and pelvic viscera preserve their functional integrity in lesions of the spinal cord. The exception of the bladder, which suffers in spinal meningitis, and softening, is only apparent, as that is an effect of paralysis of the perineal muscles.

But as we remarked before, these objections do not demonstrate an impossibility; and if Dr. Washington can prove his plan by extended, and carefully conducted experiment, the acquisition to obstetrical science will be a great one. Then shall the obstetrician, armed with a half a dozen cupping glasses, and one of Simpson's tractors for the emerging head of the *nouveau né*, go on his way rejoicing. Hour-glass uteri shall be no more, and labor shall be conducted on the principles of atmospheric pressure. S. B. H.

